# Potential Risks in the Paradigm of Basic to Translational Research: A Critical Evaluation of qPCR Telomere Size Techniques

**DOI:** 10.24218/jcet.2015.08

**Published:** 2015-08-12

**Authors:** Arthur J Lustig

**Affiliations:** Department of Biochemistry and Molecular Biology, Tulane University, USA

**Keywords:** Telomere, Telomere size, Telomere dynamics, Telomere heterogeneity, qPCR, Q-FISH

## Abstract

Real time qPCR has become the method of choice for rapid large-scale telomere length measurements. Large samples sizes are critical for clinical trials, and epidemiological studies. QPCR has become such routine procedure that it is often used with little critical analysis. With proper controls, the mean telomere size can be derived from the data and even the size can be estimated. But there is a need for more consistent and reliable controls that will provide closer to the actual mean size can be obtained with uniform consensus controls. Although originating at the level of basic telomere research, many researchers less familiar with telomeres often misunderstand the source and significance of the qPCR metric. These include researchers and clinicians who are interested in having a rapid tool to produce exciting results in disease prognostics and diagnostics than in the multiple characteristics of telomeres that form the basis of the measurement. But other characteristics of the non-bimodal and heterogeneous telomeres as well as the complexities of telomere dynamics are not easily related to qPCR mean telomere values. The qPCR metric does not reveal the heterogeneity and dynamics of telomeres. This is a critical issue since mutations in multiple genes including telomerase can cause telomere dysfunction and a loss of repeats. The smallest cellular telomere has been shown to arrest growth of the cell carrying the dysfunction telomere. A goal for the future is a simple method that takes into account the heterogeneity by measuring the highest and lowest values as part of the scheme to compare. In the absence of this technique, Southern blots need to be performed in a subset of qPCR samples for both mean telomere size and the upper and lower extremes of the distribution. Most importantly, there is a need for greater transparency in discussing the limitations of the qPCR data. Given the potentially exciting qPCR telomere size results emerging from clinical studies that relate qPCR mean telomere size estimates to disease states, the current ambiguities have become urgent issues to validate the findings and to set the right course for future clinical investigations.

## A Background of the Major Elements of Telomere Formation and Regulation

### Telomeres Structure and Function

The telomeric real-time RCR [qPCR] metric is a function of multiple aspects of telomere dynamics. This necessitates an introduction to eukaryotic telomeres. The telomere has two basic functions: terminal protection and compensation for the sequence attrition after DNA replication. Telomere DNA is composed of multiple copies of perfect or imperfect G+T-rich DNA repeats proceeding in a 5’ to 3’ direction towards the terminus. The vast majority of termini add single stranded G+T-rich DNA repeats using the ribo-nucleoprotein reverse transcriptase, telomerase, and the primase-initiated DNA polymerase a on the complementary strand. However, in cells lacking telomerase, recombination between telomeres serves as the predominant mechanism of telomere elongation and shortening [1]. Cells that utilize transposition will not be discussed in this Perspective.

Genetic studies of telomeres were initially conducted using two yeast model systems, the budding yeast Saccharomyces cerevisiae and the highly divergent fission yeast Schizosaccharomyces pombe [2–5] Biochemical studies of telomerase utilized the amplified linear DNA from ciliate macronuclei to generate a sufficiently large number of telomeres [6,7]. The catalytic subunit, telomere reverse transcriptase (TERT, yEst2), and the template-containing telomerase RNA [TR] form the core telomerase. The core telomerase is sufficient for the addition of telomere repeats onto single stranded primers in vitro [8–10] TR serves as template for telomere addition through annealing of the RNA template with single- stranded telomeric DNA. Processive telomerases stay bound to one telomere and proceeds through repetitive cycles of RNA/DNA annealing to the repeat template, telomerase extension, and translocation of the product prior to re-annealing with RNA template. Multiple repetitive cycles in cis lead to elongated telomeres containing short telomeric repeats [11]. Non-processive telomerases dissociate from the telomere and re-associate in trans with other telomeres. Repeats synthesized by either mechanism are species-specific, forming either perfect or imperfect alignment between repeats [12]. The single stranded overhang required for telomerase activity is formed by the resection of both blunt ended and the 3’ overhang telomeres by specific nucleases after replication [13,14].

In vivo, two additional proteins assist in the activation and/or binding of telomerase to the single stranded terminal 3’ overhang. The Est1 (hEst1a, hEst1b) and Est3 (hTPP1) factors bind to and facilitate the binding and activation of telomerase to form the telomerase holoenzyme [15–24]. Numerous exo-nucleases and helicases form a 3’ overhang that serves as the prime substrate for telomerase [2,25–29]. These components act redundantly and in conjunction with the single-stranded binding protein (Cdc13 in yeast) to recruit and/or activate telomerase [30,31]. The Cdt1/Stn1/Ten1 (CST) complex [32–35] acts a highly conserved regulator of the telomerase holoenzyme. While CST normally behaves as a cap, under specific conditions the subunits can also act as positive regulators of telomerase [36]. Indeed, Cdc13 is the yeast Cdt1 that acts as both positive and negative regulators of telomerase activity [37].

These activities comprise only the most basic level of regulation. Additional levels need to be considered. First, the major yeast telomere-binding protein Rap1 [repressor/activator 1] self associates and binds to an irregular telomeric repeat at a frequency of one Rap1 monomer per 20 nucleotides. This forms the core of the yeast telomere structure (or “telosome”) [38–45]. Rap1 or the telosome also serve as a barrier to end-to-end fusions mediated by non-homologous end joining [NHEJ] [46]. In humans, telomere chromatin consists of both shared and unique components termed shelterin [47,48]. The most versatile of these proteins, TRF2, appears to participate in most telomeric functions in vivo e.g. [49]. Interestingly, it binds the human homolog of Rap1. In addition, two of these components, TTP1 (yEst3) and POT1, protect the terminus from end-to end fusion [50]. The binding of POT1 is prohibited by the formation of G-quartet structures that can form in long 3’ overhangs [51].

Second, in both yeast and humans, telomeres are maintained within a genetically defined heterogeneous distribution of telomere sizes. A first-generation working model of this equilibrium has been proposed in yeast. In this model, two proteins that associate with Rap1, Rif1 [Rap1 Interacting Protein 1] [52] and Rif2 [Rap1 interacting protein 2] act predominantly as negative regulators of telomerase. Tel1 [hATM] and telomerase preferentially bind to short telomeres [53,54] while the major DDR protein Mre11/Rad50/Xrs1 (MRX) (hMRN) initiates the end resection required for the 3’ overhang. As telomeres become longer, more Rif1, recruited by Rap1, is present at higher concentrations [55,52]. Rif1 subsequently displaces Tel1 and terminates elongation. This is followed by telomere replicative attrition and, possibly, Rif1 exo-nuclease activity, leading to telomere shortening. Rif2 acts through the MRX complex to inactivate Xrs1 association with Tel1. Numerous exo-nucleases and helicases form the terminal resection that serves as the prime substrate for telomerase [2,25–29]. After multiple cell divisions, this stochastic balancing act gives rise to a genetically defined telomere distribution. An analogous feedback is present in human cells, although the components have not yet been fully elucidated [56]. The presence of heterogeneous telomeres in telomerase-negative cells was unexpected. However, this heterogeneity appears to be the result of the inheritance and maintenance of differing telomere sizes formed initially in telomerase positive cells [see [57].

Third, structural barriers to nuclease degradation and recombinases assist in telomere protection. In higher eukaryotic cells, and in at least some yeast species, telomeric loops [T-loops] appear to form as an invasion product of the 3’ terminus into distal telomeric sequences. The Holliday junction that is formed conceals the 3’ end from telomerase and deleterious activities [58,59]. After telomere resolution of the Holliday junction, C-circle products or telomere rapid deletion (TRD) truncation products accumulate. This process has been found in both fungi and human cells [60–64]. Genetic data have also revealed the presence of T-loops in budding yeast [61]. In yeast, the major telomere binding protein Rap1 interacts with the heterochromatic proteins, Sir3 and Sir4, while Sir4 interacts with the specific histone deacetylase, Sir2. These proteins in turn associate with subtelomeric de-acetylated N-termini of histones H3 and H4. The result of the multiple interactions is the formation of a subtelomeric fold back that likely facilitates the Rap1 block against NHEJ [46,65]. In addition, the G-quartet Hoogsteen base-paired structure, as noted, can form on single stranded DNA and act as a barrier to the binding of telomerase activators [66].

Fourth, the conserved TR-bound Ku70/Ku80 heterodimer acts as an inhibitor to end fusion [67]. End fusions are deleterious in most eukaryotes due to the formation of dicentric chromosomes. These dicentric chromosomes undergo repetitive fusion-bridge-breakage cycles, first identified by Barbara McClintock in wheat [68]. Fusion is dependent on a variation of NHEJ, in which telomeres or subtelomeric sequences are simply fused to one another with or without micro-homology [69].

Fifth, to cap things off, recent studies have identified the CDT1/STN1/TEN1 (CST) complex present in most eukaryotic telomeres, in addition to telosomal or shelterin protection complexes. The CST complex is a paralog of replication factor I (RFA1), and normally serves as a telomeric cap and negative regulator of telomerase [70].

### Recombinational and Replication Pathways to Telomere Formation

Other less frequent processes can result in telomere elongation. These include rolling circle replication, recombinational elongation, and gene conversion [71]. A major force in many of these processes is break-induced replication [BIR], a one-sided crossover in telomere sequences or repetitive subtelomeric sequences that is followed by fill-in synthesis using the longer complementary strand as a template. The net result is elongation of a short telomere to the size of a longer sister homolog or a non-sister chromatid [72,73]. In human cells, unequal crossing over between sister chromatids also gives rise to elongated telomeres. BIR can also preferentially elongate specific telomeres in [72]. Even under these conditions, telomere elongation is compensated by exo-nucleases and recombinational ‘telomere trimming’ as demonstrated in both normal and oncogenic human cell lines [74–76]. Similarly, in human cells, a rapid loss of sequences loss can be due to resolution of the t-loop structure followed by genome rearrangement [77–80]. Such events have been observed in primary and oncogenic cell lines. The net result is a stable equilibrium of telomere sizes.

In summary, most telomeres are formed through the negative and positive regulation of telomerase, recombination, and replication. These multiple levels of size control do not take into account the human stem cell and embryonic regulation of telomerase and recombination, tissue specific telomere size factors, and the decrease of telomere size during senescence. Mutations in the tumor suppressor proteins p53 or Rb can also overcome senescence, leading to genomic instability in yeast and humans [81–83]. The cessation of genomic instability is a strong selective force for elongation by the promiscuous activation of telomerase or by the less frequent activation of either telomere recombination. Telomere elongation subsequently produces immortalized cells and tumor formation [84,85]. The influence of telomere dynamics is difficult to gauge through just one qPCR metric. Hence, all conclusions involving qPCR telomere sizing must take into account, at least conceptually, the telomere dynamics caused by this multiplicity of activities and functions.

## Basic, Translational and Commercial Interests

The development of a rapid method for measuring telomere size in clinical and epidemiological studies has been a high priority, but requires large sample sizes (1000–2000). The required sample size precludes the use of Southern analysis as the primary technique. Large populations are needed to obtain statistical significant in telomere size changes, so that the mean telomere size as prognostic or diagnostic indicators of a disease state can be tested. However, given the complexity of telomeres and telomere regulatory pathways, the enthusiasm to correlate disease and telomere size as a diagnostic or prognostic tool must be balanced by healthy scepticism.

To overcome this sample size problem, real time PCR (qPCR) was developed to measure changes in the mean telomere size in multiple cell types at low DNA concentrations [86]. In initial studies, the primers for PCR of the telomere could anneal at multiple positions yielding a value of T. All studies contained the qPCR value of a standard gene (S). T/S values were shown to be an estimate of the true size of the telomeres as determined by Southern analysis, although significant variation was still observed. T/S value has been used as an indication of relative telomere size. A better primer construction procedure was developed during development of a multiplex qPCR. The values from this second method correlated more closely with restriction fragment lengths of telomeres [87]. In this method, one primer was constrained so that more discrete product sizes could be observed that increased the accuracy of qPCR telomere sizing relative to Southern analysis. However, whether the size of the value of the Southern blot always gives rise to the same relationship is likely to depend on the specific methods used among differing laboratories. That is, a T/S value of 1.5 may correlate with different actual sizes in independent experiments]. Similarly, small changes in the T/S metric correlated with large changes in telomere size, a barrier to accuracy [88]. These precise values varied among different laboratories. Regardless, these studies do prove a linear relationship between T/S values and the actual telomere size, although the actual alignment of the relationship may vary. Hence, with proper controls, qPCR studies can be used to measure at least relative mean telomere size.

But the problem of what the mean of a telomere size distribution by any technique signifies in a biological sense is unclear, since the real values that may be meaningful for a prognostic or diagnostic value may lie at the lower (or higher) end of the distribution. However, while the biological meaning may be unclear, a correlation with the disease state could be useful even if it is mechanistically unrelated to the disease. An important consideration is that there has been an odd tendency of many diseases to display decreases in apparent mean telomere size, raising the unusual possibility that the decreases may relate to a global stress response pathway.

The most critical analysis of the qPCR technique was a blind analysis using Cawthon’s original method conducted in two independent laboratories with experts in qPCR protocols [89]. The correlation between T/S value and telomere mean size as measured by leukocyte telomere measurements from two independent highly experienced laboratories led to a good quantitation of the variance present in the qPCR technique (CV=6%), that was, nonetheless. still lower than Southern analysis. One known source of variation, age, was identified in these studies. But most of the variation remained undefined; they might be due to small variations in technique or other factors not yet known to be involved in qPCR. Also, in some cases, the relationships were non-linear, requiring the investigators to do a significant number of experiments three times. These were blind studies using one cell type conducted by experts in the field. Other investigators contributed additional information using novel means of qPCR mean telomere size [89–95]. This study raises questions for investigators who deviate from the protocol or use different techniques. Clearly, a uniform standard method of QPCR is close as an indicator of telomere mean size, but additional studies still remain. Others have reproduced these inconsistencies that have led to conflicting data ambiguities and results.

Despite the long-term efforts of these labs to point out the limitations and provide improvements, many laboratories [*] did not universally accept some or all of these controls, leading to a non-uniform mixture of methodologies and statistical methods. If CV=6% is the best variation, the further deviations do not bode well for some of the previous data, some of which did not even compare Southern analysis with qPCR even in a subset of samples. At this point, the experiments of Aviv et al using the Cawthon method seem to be required to get close to the real changes in telomere size.

The major concern of this approach is its use in clinical and epidemiological studies prior to optimization of the procedure. The major concern is systematic error in clinical results including false positive and negative data that may lead to incorrect conclusions of major hypotheses. Nonetheless, the possibility of a technique to measure large samples sizes between disease and normal cells spread rapidly, given that the publication of such techniques in high profile journals served as non-scientific but strong validation of the procedure. The result was a plethora of studies that may be compromised by a lack reproducible data and methods [*]. We feel at the same time that basic researchers failed in their responsibility to continue to work with clinicians to place limits on the interpretation of the data. Sufficient warnings may have stemmed the tide of over-interpretation. Further damage to constraint was the quick reporting from institutions’ public relation departments and the lack of critical analysis among scientific editors who oversimplified conclusions. The lack of coordination between basic and clinical researchers was also aggravated by commercial production of qPCR telomere sizing kits that suggested to some scientists that the technique was standardized. This Perspective has two purposes: a) To inform clinicians and basic researchers about the biological variables confronting the analysis of telomere dynamics, b) To present the issues that need to be addressed for developing a universal procedure for qPCR telomere estimation. The issue is a serious attempt at compliance of uniform standards set by the community. Possibly a major advance that would allow the quick evaluation of a telomere distribution could eventually be developed.

## Procedural Variables In The Estimation of qPCR

Below are discussed the major methodological variables of the qPCR that often create difficulties in data interpretation. 1) DNA isolation, 2) Data Verification, and 3) Statistical Analysis:

### DNA Isolation

An intrinsic problem in qPCR is the potential effect of tissue type and developmental stages [96]. Three intertwined issues must be addressed: a) the basic requirements for DNA isolation, b) the nature of activities that degrade or contaminate telomere fragments in different tissues, and c) the influence of the variety of DNA isolation methodology on qPCR values.

The central requirement is to generate intact DNA from each tissue type and developmental stage [97]. Nucleases can contaminate DNA preparations, a result that is likely to vary between labs and could alter the telomere size outcome. Although it is slightly more time consuming, it is wise to use the procedure that yields more highly purified DNA. The inactivation of nucleases under PCR conditions may be a reasonable theoretical assumption, but may also be user dependent and variable. Multiple methods exist for the isolation of DNA in all eukaryotes. DNA degradation is easily identified after the addition of divalent cations such as Mg^2+^ or Mn^2+^ to an intact fragment (stored in EDTA to inhibit nucleases), and incubation at 37°C for one hour. The resulting presence or lack of nucleases should be characterized by CHEF gel electrophoresis for fragments >20 kb. For genomic samples less than 20 kb, sample can be analyzed by standard agarose gel electrophoresis. Samples that degrade after either technique should be discarded.

This suggestion is not made just out of concern for degradation at some step prior to PCR, but as an indicator of greater relevance. It is likely that nuclease contamination is proportional to the degree of overall cellular contamination. The problem, of course, is that we have no way of knowing what the activities are and to what extent they may interfere with qPCR among a) DNA preparations, b) different tissues and c) different laboratories. This simple ‘sentinel’ assay provides an essential control for the overall purity of the DNA.

### Verification of Results

Given its present experimental variability, any qPCR estimation of mean telomere size needs to be verified by a second reliable technique. This second method is, in most cases, the sizing of telomeric fragments after digestion with four base pair restriction enzymes [see below] that eliminate virtually all non-telomeric DNA fragments. Hybridization using a telomeric probe then reveals only telomeric species in Southern blots. Three major studies examined the correlation between telomere size as determined by qPCR and Southern blotting [89]. Under optimized conditions they all found strong but variable correlations. A contributing factor to this variation is the misinterpretation of Cawthon’s correlation between T/S and restriction fragment mean size as an indication that such comparisons were no longer needed in individual experiments either for a qualitative or quantitative source of data. Rather, the correlation between qPCR and Southern estimate (in a subset of samples) must be repeated in all experiments as the major validating control of qPCR mean size. Neither viewpoint was ever the intention of Cawthon or Aviv et al [who actually proved the necessity of verification for interpretation of the data. These latter studies and others also raised concern for data derived from inter-laboratory experiments even under the best of circumstances. The Southern analyses must be performed within the context of the experiment. This indicates that a random, preferably ‘blind’ sample subset should be used to test the relationship between both methods of estimating mean telomere size.

### Statistical analysis

The requirement for a consensus method of statistical analysis is absolutely essential, but has not yet been achieved. The validity of qPCR is only convincing when large sample sizes (1000–2000) produce statistically significant differences between control and experimental data, using methods that are appropriate for the qPCR technique. Given the complexity of some qPCR techniques, we recommend that a research statistician be a part of any group that is working on qPCR techniques. There should be no confusion about the statistical tests. Further, clear limits for statistical significance (at least 95% confidence or greater). Once the bar is set, all significant experiments must meet or exceed the confidence. If the data is close to significance, then additional data may just be needed to reach statistical significance. Investigators must be certain that qPCR mean sizes have been both verified and statistically significant. As recent whole genome sequence technology has entered into the telomere field, the statistical requirements have become more difficult to define and some are still being developed. A staff statistician familiar with whole genome sequence is absolutely required for the interpretation of Teloseq or Computel methods of measuring mean telomere size.

Another issue is that some hypotheses do not require sophisticated procedures to reach statistically significant see [98–100]. All investigators who work on telomere size estimates should first conduct pilot studies to measure the estimated size difference in control and experimental samples by Southern analysis. This can be used as the basis for estimating the sample size that would be needed for significance. Several labs chose qPCR on a small simple size e.g. [101]. In addition, if values have little scatter and have relatively large differences in telomere size between control and experimental groups, then standard procedures are sufficient to reach statistical significance. In addition, techniques for determination of minimal size are standard if you have initial estimates of signal and standard deviation. Researchers who have determined that a lower sample size will suffice for statistical significance may consider using Southern analysis as the standard procedure to measure telomere. In some cases, the push toward greater sophistication can introduce more variables than necessary for a relatively simple problem.

Based on the experience of yeast investigators, it is not difficult to isolate fifty DNAs from 50 small-scale cultures per day; and-contrary to popular opinion-analysis of more complex genomes need not be concerned about signal intensity. Human telomeres are reiterated and present in multiple copies and quite amenable to Southern analysis. The major variation is the use of restriction enzymes that recognize 4 bp sites. Two to four enzymes have been used to digest telomeres from adjacent or internal sequences (Rsa1, Alu1, HaeIII, Mbo1) [T. de Lange, personal communication]. After probing the digest with purified ^32^P-dCTP-labeled probes, the distribution should be equivalent to the telomere distribution. That can be formally proven by the ability of these sequences to be preferentially digested by the terminal-specific Bal31 before digestion with the restriction enzymes. Since approximately 60 digests a day can be analyzed, ‘small’ sample sizes up to 400 need not use qPCR or any more sophisticated technique.

## Telomere Characteristics That Influence qPCR Estimates

We discuss below the major biologically-based variables of the qPCR mean telomere sizing method that require qualifications in the interpretation of the qPCR T/S metric. We have already discussed many of the factors that cause telomeres to vary in size. Whether such telomere dynamics introduce the additional variables or affect interpretation of qPCR telomere mean size are discussed below. Some of these size variations have been noted in previous studies [102].

### A Lack of Isogenicity

Many experiments have been conducted to select the basic characteristics of the disease or psychological state that would minimize variation including age, ethnicity and other measurable characteristics. Most studies use multiple blood, buccal, or saliva samples]. DNA samples (extraction techniques for all is now commercially available] to determine the mean telomere size by qPCR. However, in both yeast and humans, many genes influence telomere size both directly and indirectly. In the intrinsically non-isogenic background of human genomes, several additional considerations must be taken into account.

First, non-isogenicity gives rise to a high degree of scatter of telomere size in control populations, [103]. The investigator must first determine what increase or decrease in mean telomere size of the scattered points would be statistically significant. This value would be the lower limit for a statistically significant decrease or increase in telomere size. All smaller alterations in mean telomere size cannot be used to conclude that the experimental conditions alter mean telomere size.

Second, the disease phenotype may result from a defect at a step downstream in the pathway of the disease protein. In this event, a missense, nonsense, or deletion mutation would be present in the downstream gene. Such genes may be shared among a number of differing diseases or psychologically stressed conditions. One example is the yeast Ogg1 gene (also present in humans) that operates in base excision repair of oxidized DNA from free radical activity [104,105]. But BER gives rise to shortened telomeres in yeast as well as other related phenotypes that may not be as obvious. It is also possible that differing physiologic conditions produce an alteration in scatter in the disease gene under investigation possibly through pleiotropic phenotypes. Based on the defect, investigators can deduce some different possibilities and test these experimentally.

Third, if the disease gene were involved in oncogenesis or propensity to oncogenesis, either a mutation in telomerase or in recombinational (ALT) telomere addition, a decrease in size would be predicted until the telomere became too short and dysfunctional, leading to cell cycle arrest. 15% of all tumors use the recombinational pathway. If the gene gives rise to uniform ALT cells, then methods for the accurate qPCR measurement of ALT telomeres that is well controlled would be needed. See Reddel et al for discussion of the qPCR method of sizing ALT telomeres [106].

Fourth, the gene of interest may be a driver of mutagenesis. Examples of such genes would be those involved in DNA synthesis, mismatch repair, and other DNA repair processes. The result would be a wide variety of other mutations, some of which may appear as an alteration in telomere size. Clearly any candidate for a high mutation rate must be directly tested on multiple substrates.

Fifth, although more rare, the disease state, after bypass of senescence by mutations in p53 and Rb, may be more penetrant in genes carrying shortened or elongated telomeres. Examples of this are genes involved in telomere position effects that are influenced by telomere lengths [107,108]. Genome-wide sequencing analysis, coupled with standard tests for position effects and mutagenesis, would help in drawing these conclusions. When such secondary genes cannot be ruled out, the results should nonetheless be discussed in any publication.

### The Significance of Telomere Heterogeneity on Mean Telomere Size

In mammalian cells and cell types that have a high degree of clonal heterogeneity, mean telomere size as determined by qPCR should be higher and an attempt should be made to standardize the value based upon the percentage of molecules within a size range. Since larger sized DNA having longer telomere tracts they produce greater signals in Southern analysis. In these cases, the mean telomere size must be corrected for the molecular weight at each molecular weight interval in the telomere diffuse species. These considerations follow the formula: mean telomere restriction fragment (TRF) = ΣQi/(ΣQi/ΣMWi), where Q is the signal at point i and MWi is the molecular weight at point i [109]. Although the mean is derived from a heterogeneous population of sizes, the precise relationship between mean size and heterogeneity is complex, and no simple rule can be employed to relate the two parameters. Therefore, the qPCR data must be presented accompanied by the mean, median, and spread of sizes of the telomere restriction fragments, as measured by the subset of samples using Southern analysis. Furthermore, at a theoretical level, two differing distributions could give rise to the same mean. Heterogeneity is also observed in telomerase-negative cells, but experiments by multiple labs have shown that the heterogeneity is a telomere-dependent process that is simply inherited and maintained in telomerase-negative cells.

### The Effect of Telomere Dynamics on qPCR measurements

Processes such as telomere elongation, TRD, and the formation of steady state equilibria may produce novel size classes when measured in real time. These are only observable by the behavior of telomeric species on Southern analyses. Southern blotting techniques, as the qPCR mean would undoubtedly change significantly, while the cause of the change may be unknown and possibly variable. Thus, another reason to use a subset of samples by Southern analysis is so that major rearrangements can be identified. These would be considered as outliers of the normal mean telomere size.

## The Limitations of Metadata Analyses

Metadata analyses by definition combine the data from numerous experiments. Given the inter-lab variations observed empirically, metadata is particularly prone to qPCR error. A very recent study of metadata comparisons among laboratories and among techniques came to two conclusions [110]. First, only experiments performed in a single lab can be quantified. The investigators’ second conclusion was the comparison of the methodologies and results obtained in different labs were often un-interpretable and outside the 95% confidence limits. That is, the results so far did neither confirm nor deny the conclusions. So, variables must be kept constant and never performed use data derived from prior research. This is a restriction that can probably be overcome in relatively frequent diseases or psychological states. However orphan diseases face a larger problem with small combined sample sizes The only way to avoid this problem is for consortia to use larger sample sizes provided to a centralized facility until a sufficient sample size is reached. The samples should then be tested under identical conditions. Until such a large enough sample size is accumulated, Southern analysis can be used to determine whether any major changes in telomere size are present. These data could also be used to estimate a sample size that will ultimately be required for qPCR when a sufficient number of new cases have accumulated.

## ‘New-Age’ Techniques of Large-Scale Telomere Sizing

Two newer genome-wide sequencing analysis based techniques, Teloseq [111] and Computel [112], have been introduced as a means of measuring telomere size. Both techniques are based on multiple PCR reads of telomere sequence after amplification, which typically requires a high level of significance. Studies on Teloseq involved comparisons with other methods that revealed a good correlation with actual size. Teloseq shows promise, particularly for labs engaged in high throughput whole-genome sequencing. Computel, an independent method based on similar principles, may have similar promise. However, the statistics for comparison in the multiple PCR reactions needed for genome-wide sequencing remain complex and are beyond the abilities of most investigators’. Thus, any such study should have a qualified research statistician as part of the team. Over time, these problems will presumably be solved. Investigations should still rely on internal controls for verification of the mean average size as used in the Teloseq study.

## Co-Fish As An Alternative Measure of Telomere Size and Dysfunction

In case of ambiguous qPCR results that are not resolved by Southern analysis, in situ quantitative FISH [Q-FISH; techniques provide an alternative independent method of estimating telomere size and function This may be valuable, since fusions (the products non-functionally short telomere) are directly related to telomere dysfunction. Telo Q-FISH [113,114] can determine the presence of dimers, and can also define the length if appropriate molecular weight telomere standards are included. Since the loss of a single telomere leads to cell arrest [115,116], and, conversely, uncontrolled growth of elongated telomeres in Tetrahymena lead to “enlarged monster cells”, a clearer physiological defect at the telomere can be ascertained using microscopy. The particular success of the newly described method of ‘high throughput Co-FISH’ is best alternative to identify aberrant function and even to rule out artifacts of qPCR [117,118].

When used in the absence of qPCR for genes giving rise to oncogenic states, this method can more accurately measure the higher rate of fusions and dicentric chromosomes in mitosis that lead to chromosome loss and cell death. Using high throughput alternatives of Telomeric-FISH, larger groups of disease genes sizes can be analyzed in the absence of any intrinsic bias.

## Summary/Looking Back and Ahead

What are the most important uncontrolled factors that are required for the current analysis of qPCR. Several variations including the purity of the DNA, the comparison of identical tissue types and physiological states and the stability of the telomere in vitro are given requirements. What is also critical is that investigators do not produce conclusions that exceed the limits of the data. Caveats are present in any technique. Investigators are aware of the limitations of their studies and should present them within publications in a transparent fashion. A site should be developed for both significant negative and positive data with all relevant methodology, so that wasteful repeated efforts by other labs can be avoided.

One of the most critical experimental issues from qPCR is an increased degree of consistently with control data, allowing qPCR data to be a more exact measure of mean telomere size. This outcome, of course, depends on the compliance of individuals within the field and within the peer review process to use the same criteria. With greater compliance in a uniform procedure, the relative accuracy of the mean telomere size should become less error prone.

One central conceptual issue is the relationship of statistical significance of qPCR mean telomere size to biological significance. A clarification of this issue will lead the way to newer measurements of the heterogeneity and extremes of telomere sizes and their relationship to the disease phenotype. This will be possible if the disease states are caused by specific genetic mutations or epigenetic characteristics. Further experiments will be needed to establish a causal relationship in well-characterized disease genes and mean telomere size. The multiple factors that may be at play in psychological syndromes are more difficult to determine in a causal fashion unless specific genes can be identified that are related to the behavior.

A second critical conceptual issue is whether the analysis of qPCR mean telomere size (or the distribution of sizes) indicate whether a correlation with multiple disease states, living conditions and psychological states reflects reflect the direct effects of the mutant genes or is the consequence of stress-response pathways (Reiter et al., 2008). An examination of the behavior of stress response genes under disease conditions should be a fascinating study that may relate telomere size changes to stress response genes. This rather interesting possibility cannot be ascertained at present and is certainly a topic for a Perspective in coming years.
